# Concurrent clinical and pathological response predicts favorable prognosis of patients with gastric cancer after neoadjuvant therapy: a real-world study

**DOI:** 10.1186/s12885-023-11508-8

**Published:** 2023-10-18

**Authors:** Chongyuan Sun, Penghui Niu, Xiaojie Zhang, Lulu Zhao, Wanqing Wang, Xiaoyi Luan, Xue Han, Yingtai Chen, Dongbing Zhao

**Affiliations:** National Clinical Research Center for Cancer/Cancer Hospital, National Cancer Center, Chinese Academy of Medical Sciences and Peking Union Medical College, Beijing, China

**Keywords:** Gastric cancer, Neoadjuvant therapy, Prognosis, Pathological response, Clinical response

## Abstract

**Background:**

Response of locally advanced gastric cancer (LAGC) to neoadjuvant therapy (NAT) may be associated with prognosis, but which of the clinical or pathological evaluation can accurately predict a favorable prognosis is still controversial. This study aims to compare the effect of clinical and pathological response on the prognosis of patients with gastric cancer.

**Methods:**

This study retrospectively analyzed LAGC patients who underwent NAT followed by surgery in the China National Cancer Center from January 2004 to January 2021. Clinical and pathological responses after NAT were evaluated using RECIST 1.1 and Mandard tumor regression grade system (TRG) respectively. Complete response (CR) and partial response (PR) assessed by computed tomography were regarded as clinical response. For histopathology regression assessment, response was defined as Mandard 1, 2, 3 and non-response as Mandard 4, 5. Furthermore, we combined clinical and pathological evaluation results into a variable termed “comprehensive assessment” and divided it into four groups based on the presence or absence of response (concurrent response, only clinical response, only pathological response, both non-response). The association between the prognosis and clinicopathological factors was assessed in univariate and multivariate Cox regression analysis.

**Results:**

In total, 238 of 1073 patients were included in the study after screening. The postoperative pathological response rate and clinical response rate were 50.84% (121/238) and 39.92% (95/238), respectively. 154 patients got consistent results in clinical and pathological evaluation (66 were concurrent response and 88 were both non-response), while the other 84 patients did not. The kappa value was 0.297(p < 0.001), which showed poor consistency. Multivariate Cox regression analysis revealed that comprehensive assessment (P = 0.03), clinical N stage(P < 0.001), vascular or lymphatic invasion (VOLI) (HR 2.745, P < 0.001), and pre-CA724(HR 1.577, P = 0.047) were independent factors for overall survival in patients with gastric cancer. Among four groups in the comprehensive assessment, concurrent response had significantly better survival (median OS: 103.5 months) than the other groups (P = 0.008).

**Conclusion:**

Concurrent clinical and pathological response might predict a favorable prognosis of patients with gastric cancer after neoadjuvant therapy, further validation is needed in prospective clinical trials with larger samples.

## Introduction

Gastric cancer (GC) is the fifth most frequently diagnosed cancer and the fourth leading cause of cancer death worldwide [1]. As typical symptoms and screening systems for early gastric cancer are lacking, about 70% of patients with GC were diagnosed with locally advanced disease [2]. Currently, neoadjuvant therapy (NAT) followed by surgery is the standard care of treatment for locally advanced gastric cancer (LAGC) patients, due to its benefits for eliminating micrometastases, down-staging tumor burden and boosting chance of curative resection [3, 4].

There are various methods available for evaluating the efficacy of NAT for gastric cancer at present. The Response Evaluation Criteria in Solid Tumors (RECIST) is the most commonly utilized set of criteria for appraising tumor response to NAT [5]. Additionally, in order to evaluate tumor response histologically following therapy, the tumor regression grade (TRG) is frequently used [6]. Regarding pathological evaluation, numerous studies have indicated that pathological response portends a favorable prognosis, particularly in terms of pathological complete response (pCR) [7, 8]. However, in clinical practice, the results of the two evaluation criteria may exhibit discrepancies [9, 10]. Some studies have investigated the incongruity between the two sets of criteria and have explored the prognostic value of clinical and pathological response separately instead of in combination [11, 12]. This approach may result in a less objective and accurate appraisal of treatment response.

As such, we conduct this study and enroll GC patients following NAT to assess the prognostic value of combining clinical evaluation (RECIST) with pathological evaluation (Mandard-TRG) and screen the clinicopathological factors associated with prognosis.

## Methods

### Patient selection

The study queried data from a high-volume GC cohort at China National Cancer Center, encompassing more than 1,000 GC patients. The demographic information, clinicopathological features, pre-treatment serum tumor biomarkers (CA724, CEA), pre-and post-treatment imaging, and gross specimens after gastrectomy of GC patients who received NAT between January 2004 and December 2021 were retrospectively retrieved. The main inclusion criteria consisted of the following: (1) confirmation of primary gastric adenocarcinoma through pathological examination; (2) administration of NAT prior to gastrectomy; (3) locally advanced gastric carcinoma without distant metastasis (clinical TNM stage: cT2 ~ T4 and cN0 ~ N3, II–III); and (4) radical surgical excision + D2 lymph node dissection. The main exclusion criteria were as follows: (1) presence of other malignant neoplasms; (2) inoperable or non-resected cases; and (3) lack of follow-up or missing response data to NAT. Finally, 238 GC patients were identified based on these screening criteria. The pre-therapeutic clinical staging (cTNM) within this study was assessed using the American Joint Committee on Cancer (AJCC) TNM 8th edition staging system.

### Evaluation system for clinical and pathological response

Based on computed tomography (CT) scans, two professional radiologists evaluated the clinical response according to the RECIST version 1.1 criteria. Target lesions were defined as either the primary tumor with a maximum diameter greater than 10 mm or enlarged lymph nodes with a short axis greater than 15 mm [5]. The response categories were as follows: CR (complete response) means the complete disappearance of all target lesions, PR (partial response) means the sum of the diameters of all targets lesions decreases ≥ 30%, PD (progressive disease) means the sum of the diameters of all targets lesions increases ≥ 20%, SD (stable disease) means insufficient shrinkage to qualify for PR or insufficient increase to qualify for PD). Patients who achieved either CR or PR were classified as having a clinical response.

Histologic regression was evaluated using the Mandard TRG as follows: TRG 1 = Complete regression or fibrosis with no evidence of tumor cells; TRG 2 = Fibrosis and rare residual cancer cells; TRG 3 = Fibrosis outgrowing residual cancer; TRG 4 = Rare fibrosis and Residual cancer outgrowing fibrosis; TRG 5 = Tumor without evidence of regressive changes [6]. The Mandard TRG score was assessed by 2 independent pathologists affiliated with the department of pathology at our hospital. In the present study, pathological response was defined as TRG 1–3, while TRG 4 and 5 were classified as non-response. Furthermore, we introduced a synthetic variable termed “comprehensive assessment” and patients were divided into 4 groups based on their responses in clinical and pathological evaluation: concurrent response, only response in TRG, only response in RECIST 1.1, and both non-response.

### Tumor markers

The levels of CEA and CA72-4 were obtained through laboratory analysis of the patient’s routine blood test at the time of initial diagnosis with upper normal values of 5 ng/mL and 6.9 ng/L, respectively. The optimal cutoff values for pre-CEA and pre-CA72-4 were determined using the ‘surv-cutpoint’ function of R package ‘survminer’. The cutoff values for pre-CEA and pre-CA72-4 were found to be 7.29 ng/mL and 10.27 ng/mL respectively.

### Statistical analysis

SPSS ver. 26.0 software (IBM Corp., Armonk, NY) was used to perform statistical analysis in this study. Categorical variables were presented as counts and percentages. Overall survival (OS) was defined as the time from the initial treatment to death by any cause or the final follow-up. The OS curves were compared using the log-rank test among different evaluation criteria. Cox regression analysis was utilized to assess the hazard ratios of all factors for OS, and the factors with P value ≤ 0.1 or of significant clinical importance were included in the multivariable analysis. A p-value less than P < 0.05 was considered statistically significant and all tests were two-sided.

## Result

### Patients characteristics

**Fig. 1:**
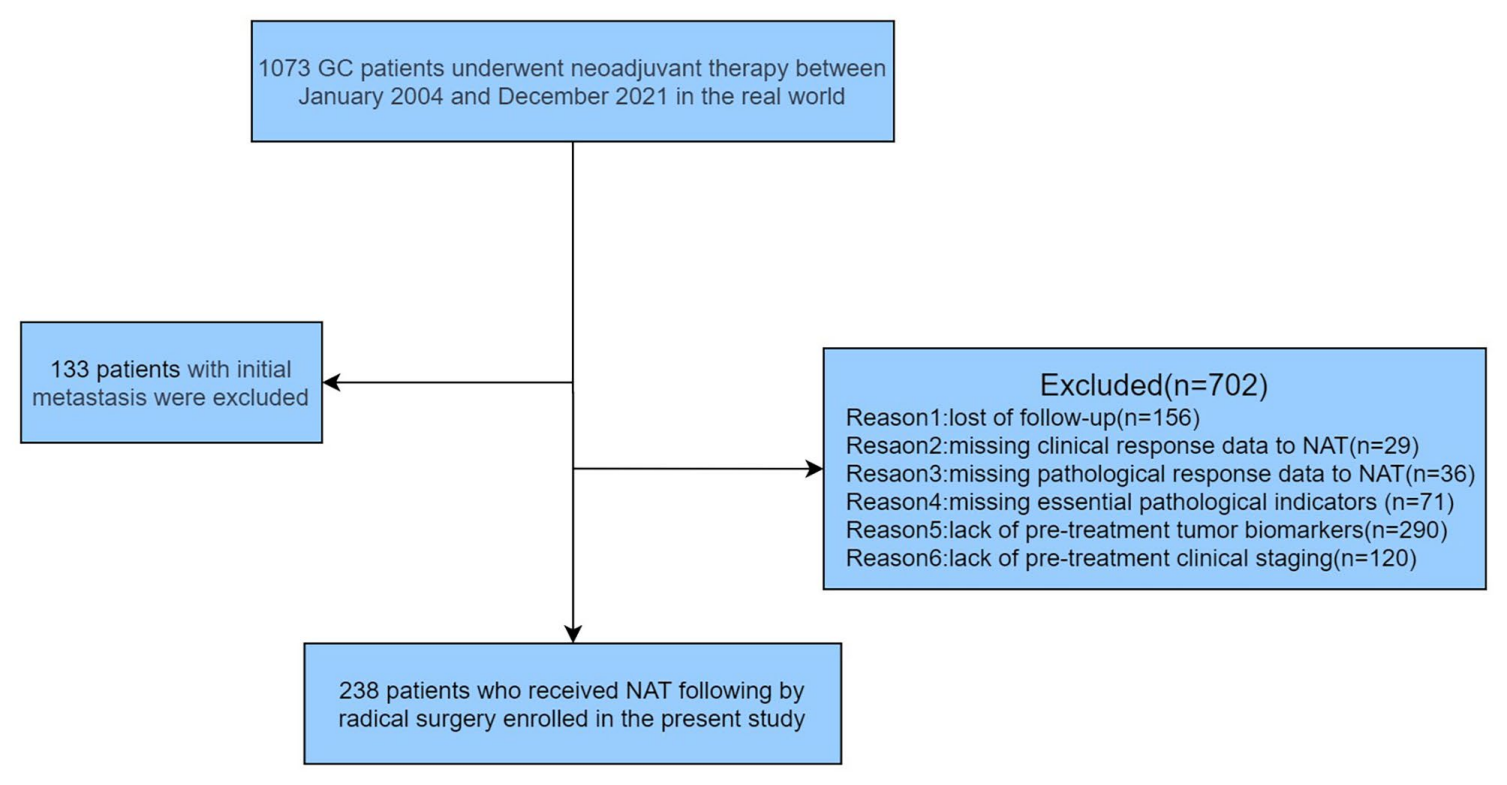
Flow diagram illustrating recruitment of gastric cancer patients

A total of 1073 patients receiving NAT between January 2004 and December 2021 were screened for inclusion in this study. 133 patients with initial metastasis such as liver metastasis or peritoneal metastasis were excluded from the study. Finally, 238 patients met the inclusion and exclusion criteria and were included in the result analysis (see Fig. 1). Table 1 reports the baseline demographics and clinical characteristics of GC patients. The majority of patients were male (77.7%), and the median age was 59 years old (ranging from 22 to 84). Ninety cancers were located at the gastric cardia or the fundus (37.8%), while most primary tumors (53.3%) were located at the gastric body or antrum. In 21 cases, GC involved three segments of the stomach (linitis plastica). Seven patients (7/114, 6.1%) were identified as mismatch repair deficient (dMMR), and 12 patients (12/201, 6.0%) were human epidermal growth factor receptor-2 (HER-2) positive. In terms of NAT regimens, the majority of patients (92.0%) received neoadjuvant chemotherapy, with oxaliplatin combined with S-1/capecitabine regimens(53.0%) and docetaxel plus oxaliplatin and S-1/capecitabine regimens(34.7%) being the most commonly used chemotherapy regimens. Additionally, 8.0% of the patients underwent neoadjuvant concurrent chemoradiotherapy. For these patients, a total dose of 45 Gy was applied, using 25 fractions of 1.8 Gy within 5 weeks delivered concurrently with S-1 at 80 mg/m^2^.

**Table 1:** Demographic and clinicopathologic characteristics of gastric cancer patients following neoadjuvant therapy(n = 238)

Characteristic	No.of patients	Percent(%)
Gender		
Male	185	77.7
Female	53	22.3
Age (years)		
<60	141	59.2
≥60	97	40.8
BMI(kg/m2)		
< 23.9	135	56.7
≥ 23.9	102	42.8
Unknown	1	0.5
ASA score		
1	13	5.4
2	205	86.1
3	16	6.7
Unknown	4	1.8
Tumor location		
Upper	90	37.8
Lower	127	53.3
Diffuse	21	8.9
Tumer size(cm)		
< 2	23	9.6
2-5	133	55.9
≥ 5	82	34.5
Surgical type		
Proximal	66	27.7
Distal	100	42.0
All stomach	72	30.3
cT		
2	7	2.9
3	49	20.6
4	182	76.5
cN		
0	32	13.5
1	73	30.7
2	76	31.9
3	57	23.9
Lauren classification		
Intestinal or Mixed	93	39.1
Diffuse	82	34.4
Unknown	63	26.5
Grade of differentiation		
Well	3	1.3
Moderate or Poor	219	92.0
Unknown	16	6.7
Vascular or lymphatic invasion		
No	169	71.0
Yes	69	29.0
Nervous invasion		
No	129	54.2
Yes	109	45.8
NAT pattern		
Chemotherapy	219	92.0
oxaliplatin+S-1/capecitabine	116	53.0
docetaxel+oxaliplatin+S-1/capecitabine	76	34.7
Paclitaxel+oxaliplatin	6	2.7
Paclitaxel+S-1	5	2.3
S-1	2	0.9
Other/unknown	14	6.4
Concurrent chemoradiation	19	8.0
Cycle of NACT		
< 5	208	87.4
≥ 5	30	12.6
Adjuvant chemotherapy		
Yes	185	77.7
oxaliplatin+S-1/capecitabine	118	63.8
docetaxel+oxaliplatin+S-1/capecitabine	36	19.4
Paclitaxel+oxaliplatin	9	4.9
Paclitaxel+S-1	10	5.4
S-1	7	3.8
Other	5	2.7
No	6	2.6
Unknown	47	19.7

BMI Body Mass Index, ASA score American society of Aneshesiologists(ASA)physical status classification system, NACT neoadjuvant chemotherapy

The TRG scores were as follows: TRG 1 (n = 26, 10.9%); TRG 2 (n = 18, 7.6%); TRG 3 (n = 77 32.4%); TRG 4 (n = 39, 16.4%); TRG 5 (n = 78, 32.7%). The number and proportion of patients with different clinical evaluation results were as follows: PR group (n = 95, 39.9%), SD group (n = 134, 56.3%), and PD group (n = 9, 3.8%). The median number of cycles of neoadjuvant chemotherapy was 4, and 185 patients (185/191, 77.7%) received adjuvant chemotherapy after surgery.

### Consistency analysis of clinical and pathological evaluation

In this study, TRG 1–3 were considered as response, while TRG 4 or 5 were classified as non-response. Similarly, RECIST CR or PR were considered as response, while SD or PD were classified as non-response. Of the total patient population, 154 patients had consistent results in both clinical and pathological evaluation (66 patients had concurrent responses, and 88 had both non-responses). However, 29 patients had a response in clinical evaluation but not in pathological evaluation, while 55 patients had a response in pathological evaluation but not in clinical evaluation. The kappa value, a measure of agreement between the two evaluation methods, was 0.297 (p < 0.001), indicating poor consistency between clinical and pathological evaluations.

### Survival in different groups

**Fig. 2:**
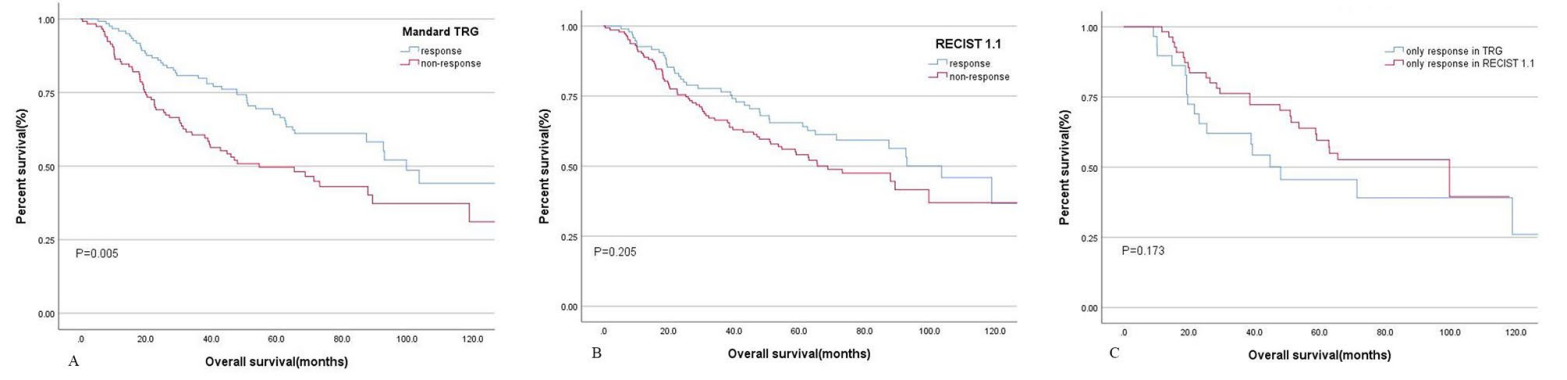
Kaplan-Meier curves for different evaluation criteria. **A**. Overall survival of Mandard TRG response group and Mandard TRG non-response group; **B**. Overall survival of RECIST response group and RECIST non-response group; **C**. Overall survival of only clinical response group and only pathological response group

The median survival time for patients in different groups was presented in Table 2. In all patients, the median survival times were 103.5, 73.1, and 38.5 months for PR, SD, and PD groups, respectively. The Mandard 1–3 and Mandard 4–5 groups had median survival times of 99.6 and 54.6 months, respectively. Among the four groups of comprehensive assessment, the median survival times for concurrent response, only pathological response, only clinical response, and both non-response were 103.5, 99.6, 48, and 65.3 months, respectively. Survival analysis was conducted on different evaluation criteria and the log-rank test showed a significant difference among groups within Mandard TRG (P = 0.005) and comprehensive assessment (P = 0.022). Patients who were only responsive in TRG tended to have a better prognosis than those who were only responsive in RECIST, though the difference was not statistically significant (99.6 months vs. 47.9 months for OS, P = 0.173) (Fig. 2).

**Table 2:** Median survival time in patients with different evaluation criteria

Evaluation criteria	Category	N0.(percent)	Median survival(months)	Log-rank test
RECIST 1.1				P=0.205
	PR	95(39.9)	103.5	
	SD	134(56.3)	73.1	
	PD	9(3.8)	9.4	
Mandard TRG				**P=0.005**
	1-3	121(50.9)	99.6	
	4-5	117(49.1)	54.6	
Comprehensive assessment				**P=0.022**
	both non-response	88(37.0)	65.3	
	only clinical response	29(12.2)	48.0	
	only pathological response	55(23.1)	99.6	
	both response	66(27.7)	103.5	

RECIST 1.1 Response Evaluation Criteria In Solid Tumours 1.1, Mandard TRG Mandard tumor regression grade

The study included clinicopathological features, such as age, sex, tumor size, site of tumor, BMI, vascular invasion or lymphatic invasion, comprehensive assessment, etc. to conduct univariate analysis and identify prognostic factors for OS (Table 3). The results showed that Lauren classification, cycle of NAT, nervous invasion, pre-CA724, tumor size, vascular invasion or lymphatic invasion, clinical N (cN) stage, clinical T (cT) stage, and comprehensive assessment were related with OS on univariate analysis. Furthermore, multivariable Cox proportional hazard model analysis that included the aforementioned risk factors showed vascular invasion or lymphatic invasion, cN stage, pre-CA724, and comprehensive assessment were significantly associated with OS (vascular invasion or lymphatic invasion: P < 0.001; cN stage: P < 0.001; pre-CA724: P = 0.047; comprehensive assessment: P = 0.030, Table 3).

**Table 3:** Univariate and multivariate analyses for overall survival using a Cox proportional hazards model

Variables	OS			
	Univariate		Multivariate	
	Hazard ratio	P	Hazard ratio	P
Age (years)				
≤ 60	1			
> 60	0.861(0.650,1.141)	0.298		
BMI (kg/m2)				
≤ 23.9	1			
> 23.9	0.844(0.638,1.116)	0.235		
Gender				
Male	1			
Female	1.262(0.931,1.711)	0.133		
Tumor location		0.363		
Upper	1			
Lower	1.206(0.896,1.623)	0.216		
Diffuse	0.928(0.530,1.625)	0.794		
Surgical type		0.269		
Proximal	1			
Distal	0.865(0.619,1.209)	0.396		
All stomach	1.135(0.797,1.616)	0.484		
Tumer size(cm)		**<0.001**		0.129
≥ 5	1		1	
< 2	0.357(0.202,0.630)	**<0.001**	0.843(0.358,1.987)	0.697
2-5	0.574(0.428,0.771)	**<0.001**	0.652(0.431,0.988)	**0.044**
Lauren classification				
Intestinal or Mixed	1		1	
Diffuse	1.421(1.033,1.954)	**0.031**	1.088(0.694,1.706)	0.712
cT		**0.002**		0.142
4	1		1	
3	0.487(0.324,0.731)	**0.001**	0.574(0.312,1.058)	0.075
2	0.564(0.209,1.524)	0.259	0.348(0.046,2.664)	0.310
cN		**<0.001**		**<0.001**
3	1		1	
0	0.350(0.219,0.559)	**<0.001**	0.529(0.245,1.141)	0.104
1	0.299(0.204,0.439)	**<0.001**	0.275(0.162,0.468)	**<0.001**
2	0.370(0.258,0.530)	**<0.001**	0.417(0.248,0.702)	**0.001**
Grade of differentiation				
Moderate or Poor	1			
Well	0.949(0.446,2.019)	0.893		
Vascular or lymphatic invasion				
No	1		1	
Yes	2.779(2.103,3.672)	**<0.001**	2.745(1.688,4.463)	**<0.001**
Nervous invasion				
No	1		1	
Yes	1.664(1.258,2.202)	**<0.001**	0.844(0.505,1.409)	0.517
Adjuvant chemotherapy				
Yes	1			
No	0.600(0.191,1.885)	0.382		
Cycle of NACT				
≤ 5	1		1	
> 5	1.644(1.121,2.411)	**0.011**	1.411(0.812,2.450)	0.222
pre-CA724				
≤10.27	1		1	
>10.27	2.339(1.614,3.389)	**<0.001**	1.577(1.007,2.470)	**0.047**
pre-CEA				
≤7.29	1		1	
>7.29	1.500(1.037,2.168)	**0.031**	1.480(0.941,2.327)	0.089
comprehensive assessment		**<0.001**		**0.030**
both non-response	1		1	
only clinical response	0.887(0.583,1.350)	0.576	1.149(0.647,2.038)	0.636
only pathological response	0.803(0.560,1.152)	0.233	0.846(0.496,1.445)	0.541
both response	0.417(0.292,0.597)	**<0.001**	0.480(0.280,0.822)	**0.008**

BMI Body Mass Index, NACT neoadjuvant chemotherapy, pre-CA724 pre-treatment carbohydrate antigen199, pre CEA pre-treatment carcinoembryonic antigen

**Fig. 3:**
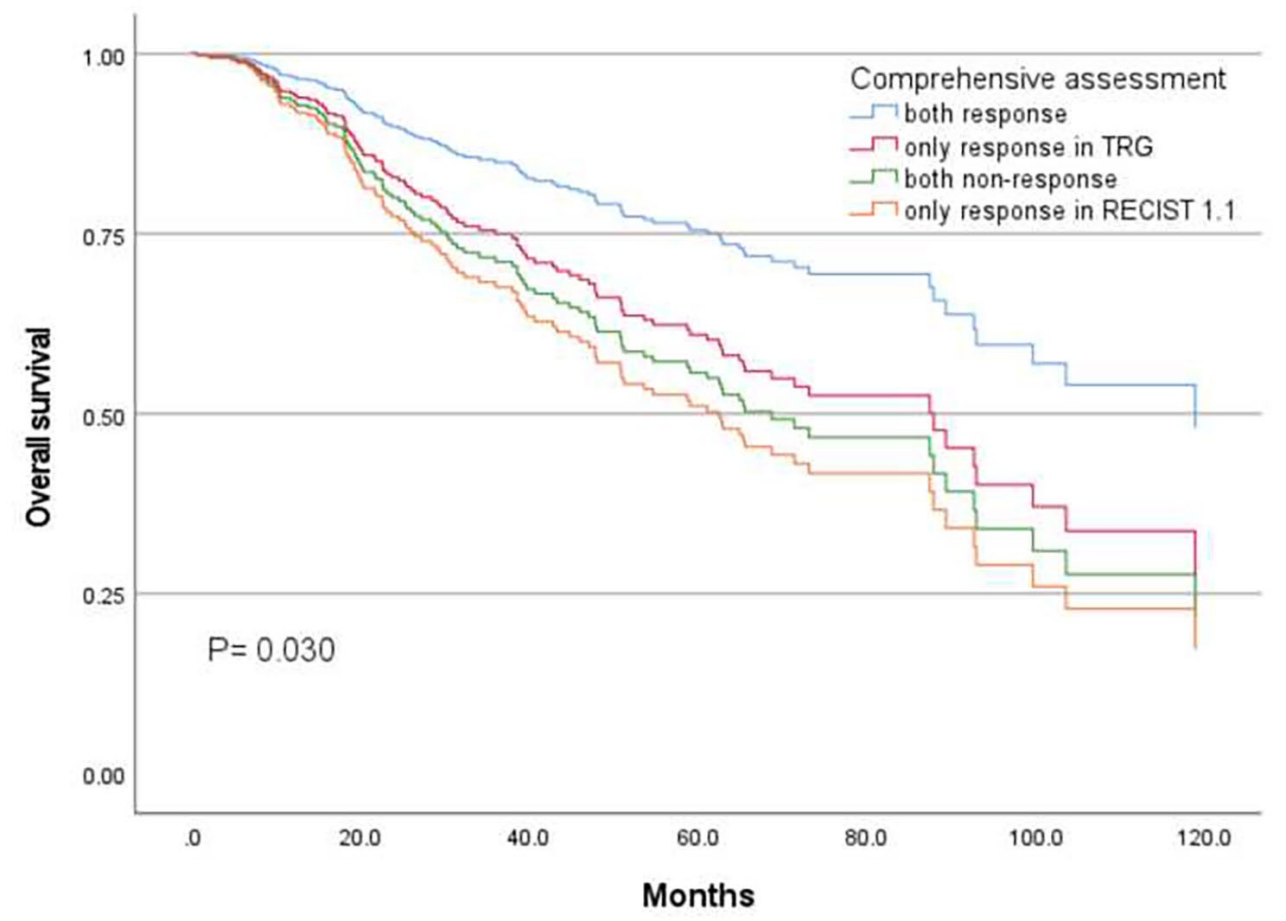
Kaplan-Meier curve of comprehensive assessment in the multivariate analysis

After including clinical and pathological evaluation as a variable termed comprehensive assessment in the model, only the concurrent response group had a significantly better survival (median OS: 103.5 months) than the other groups (P = 0.008) (Fig. 3).

## Discussion

This real-world study enrolled 238 gastric cancer patients following NAT from National Cancer Center in China over the past fifteen years and assess the prognostic value of clinical evaluation (RECIST) combined with pathological evaluation (Mandard-TRG). It demonstrated poor consistency between pathological and clinical evaluations, and suggested that concurrent clinical and pathological response might predict a favorable prognosis in patients with GC. Besides, vascular invasion or lymphatic invasion, cN stage, and pre-CA724 were significantly associated with prognosis.

The assessment of NAT efficacy plays a crucial role in deciding postoperative chemotherapy regimens for LAGC patients. However, there is currently no unified standard for evaluating neoadjuvant treatment response and many authors have proposed various methods, including pathological tumor regression assessed by the TRG system, radiological tumor regression according to RECIST version 1.1 and serum tumor marker [5, 6, 13]. A retrospective study conducted by Wang et al. found that TRG (P = 0.042, HR = 1.65) was an independent prognostic factor affecting the OS of GC patients, and patients who had a pathological response to NAT had a better prognosis [14]. Pietro et al. analyzed 67 LAGC patients who had received preoperative chemotherapy and found that clinical response was a prognostic factor for both OS and DFS (OS:P = 0.003; DFS: P = 0.003) [9]. Similar conclusions have been reached in many studies [15, 16]. However, the results of our study indicate that the evaluation results of clinical and pathological responses are not always consistent. To address this issue, we introduced a new variable called comprehensive assessment which synthesizes the evaluation criteria of different dimensions, and divided patients into 4 groups according to their responses in TRG and RECIST 1.1. Multivariate Cox regression analysis confirmed that comprehensive assessment was significantly associated with the prognosis of LAGC, and only the concurrent response group showed a significant difference when compared with the non-response group. This highlights the importance of considering both clinical and pathological responses in evaluating NAT efficacy and suggests that a comprehensive assessment may provide a more accurate and reliable prognostic evaluation for LAGC patients. In addition, the lack of statistical difference between patients with only pathological response and those with only clinical response could be due to the association of allelic imbalance at markers of the HLA region with decreased survival, which was observed only in patients with pathological response and not in those without pathological response [17].

Despite being widely applied in NAT assessment and considered as the clinical evalution standard in assessing tumor regression response for some solid malignancies, the Response Evaluation Criteria in Solid Tumors (RECIST v1.1) did not demonstrate a significant association with clinical outcomes in our study, which was inconsistent with pathological evaluation. The possible reasons may include: firstly, the fibrosis, necrosis, or edema of tumor tissue after chemotherapy may distort the layers of the stomach, affecting the evaluation of the residual tumor size [18]; Secondly, clinical evaluation requires a measurable lesion which may not suitable for some lesion originating from digestive tract [5, 19], especially poor stomach filling on CT images. Thirdly, different imaging devices and measurement errors might also contribute to the inconsistency. However, pathological evaluation results are based on postoperative pathological findings, making it impossible to assess tumor regression in real time to guide treatment. Therefore, novel methods for clinical evaluation were warranted to more accurately predict the response to NAT. A retrospective study collected 1231 radiomic features from CT images of 292 LAGC patients and found the detection radiomics (DR) model based on 28 cross-combination models was superior to the commonly used RECIST method (NRI 39.5% and NRI 35.4%) [20]. Additionally, total iodine uptake of portal phase (TIU-p) was found to improve the accuracy of pathological evaluation in advanced gastric cancer patients after neoadjuvant chemotherapy (r = 0.602, P = 0.000) [21]. Moreover, the volume reduction rate (VRR) calculated by tumor volume changes before and after NAT was also found to be a feasible and reliable method to assess the histopathologic tumor response [22].

Serum tumor markers are widely used in the diagnosis, prognostic prediction, and recurrence monitoring of gastrointestinal malignancies. Some studies have demonstrated that CA724 is related to the pathological stage and has an excellent diagnostic value for GC [23, 24]. In our research, CA724 prior to NAT was identified as an independent prognostic factor through multivariable analysis which is in agreement with the findings of Sun et al. [25]. Similarly, Tong et al. found CA724 prior to treatment was an independent risk factor to pathological reaction which is beneficial to predict TRG [26]. Lymph node metastases, invasion depth and vascular invasion or lymphatic invasion are independent prognostic indicators of survival in patients with GC [27]. Our study demonstrates that vascular invasion or lymphatic invasion, cN were significantly associated with survival, which was coincident with previous ones [28]. Unfortunately, cT stage failed to be an independent factor for OS in patients after NAT. This may be due to a relatively high proportion of cT4 cases and poor accuracy of CT scan in T staging evaluation [29]. Wang et al. reported that the cT stage does not impact overall survival in GC patients who accepted NACT treatment. From this point, our study was in line with Wang’s study, showing T stage was not independent factors for survival in patients after perioperative chemotherapy [14].

There were some limitations in our study. Firstly, this retrospective study is subject to selection bias due to inherent limitations in sample selection and data collection. Secondly, the regimens for preoperative treatment were different. Finally, some factors, including comorbidities and gene signatures, were not enrolled in our study. Despite the limitations above, we revealed the relationship between clinical evaluation (RECIST 1.1) and pathological evaluation (Mandard TRG) directly, which was rare in previous studies. Furthermore, we innovatively incorporated clinical and pathological evaluation into a single variable and focused on exploring the impact of inconsistent evaluation results on prognosis.

## Conclusion

This real-world study demonstrated that concurrent clinical and pathological response might predict a favorable prognosis for patients with GC, whereas a single clinical or pathological response could not if they are contradictory. Moreover, pathological assessment (TRG) was in poor agreement with clinical assessment (RECIST 1.1), and cN stage, vascular or lymphatic invasion and pre-CA724 were identified as independent factors for OS in LAGC patients. Further studies with a larger sample size are needed to confirm our findings.

## Data Availability

The datasets used and/or analyzed during the current study are available from the corresponding author on reasonable request.
